# Supplementary material for: Anomaly based multi-stage attack detection method
Source: PLoS One. 2024 Mar 25;19(3):e0300821. doi: 10.1371/journal.pone.0300821 (PMC10962843; doi:10.1371/journal.pone.0300821)
Supplement: Attachment — Submitted filename: Response-to-Reviewers.docx [file pone.0300821.s001.docx]

**Original Article Title: “Anomaly based multi-stage attack detection method**”

**R****eviewer#1, Concern # 1:** The main contribution and originality should be explained in more detail; why is it important?

**Author response:** Thank you very much for your valuable comments. We have modified the manuscript to describe the specific contributions in detail in the first section, highlighting the innovations of this paper.

…

Compared to previous methods, our approach can extract deep-seated information from alerts, analyze the relationships between alert entries, and accomplish model building without the need for prior knowledge using unsupervised clustering methods.

The main contributions of this paper are as follows:

- An original concept of Multi-Stage Profile (MSP) was introduced. Borrowing from the idea of dividing attacks into multiple stages, the paper performs staged modeling of a system's normal state. This enhances the model's adaptability and generalization to different network environments and attack types, improving its overall adaptability and generalization.
- To accommodate various attack scenarios, a novel method for adaptive acquisition stages is proposed. This method utilizes Doc2Vec to deeply extract information from alert messages, preserving the interrelations between alerts more effectively. Subsequently, it clusters vectorized alert information, grouping semantically similar alerts into the same stage. This method demonstrates adaptability to various attack scenarios.
- An original concept of Generating Probability (GP) is introduced, used to calculate the probability of an alert sequence occurring. We employ GP to establish anomaly thresholds, enabling attack detection.
- Comparative experiments conducted on three different datasets (DARPA2000, ISCXIDS2012, CIC-IDS2017): Extensive experimental results illustrate that this method achieves an accuracy of over 99\% in detecting multi-stage attacks across various datasets, outperforming the latest methods.

**Reviewer#1, Concern # 2:** More comparison of results against alternative approaches is needed for the benefit of the readers.

**Author response:** Thank you very much for your valuable comments. Some more detailed analyses of the results are given in the Analysis of experimental results section of the manuscript to express that the performance of our method is better than the alternative approaches. And we also made a discussion to explain the reasons behind the results. The modifications are as follows:

…

From Table5, 6, and 7, it can be observed that on all three datasets, whether it is Accuracy, Precision, Recall, or the comprehensive F1-score, our method exhibited the best performance. On the DARPA2000 dataset, our method achieved 99.2% Accuracy and 100\% Precision, with an F1-score of 98%, surpassing Pre-HMM, which also utilizes HMM. The deep learning methods Seq2Seq and LSTM had F1-scores of 93% and 94.8%, slightly lower than our approach. On the ISCXIDS2012 dataset, our method attained 100\% across all four metrics. Seq2Seq and LSTM both had F1-scores above 90\%, with Seq2Seq performing better. Pre-HMM still exhibited the poorest performance with an F1-score of only 60.9%. For the CICIDS2017 dataset, our method achieved an F1-score of 98.1%, followed by Seq2Seq at 95.2%, LSTM at 89%, and Pre-HMM at the lowest with 87.3%. According to our analysis, the main reasons for this result are as follows: First, we use doc2vec to vectorize the alarm text, which is more conducive to capturing the deep information of the alarm information and mining the internal correlation between each other, so the performance is obviously better than the Pre-HMM using word2vec; Secondly, our method is mainly to find abnormal conditions in the alarm sequence without specifically detecting the attack stage, so the accuracy is high. Lastly, multi-stage attack detection is not time-sensitive, which is why Seq2Seq outperforms LSTM on all datasets as a deep learning method.

**Reviewer#1, Concern # 3:** Authors should argue their choice of performance evaluation indicators.

**Author response:** Thank you very much for your valuable comments. We have explained the reasons for selecting the evaluation criteria in the Evaluation Indicators section, and the specific changes are as follows:

…

In the evaluation task, the most commonly used model evaluation metrics are accuracy, precision, recall, and F1-score. These metrics provide comprehensive insights into the model's performance, particularly in classification tasks. Therefore, this paper selects these four metrics as the model evaluation criteria, and these metrics are calculated using the following formulas.

**Reviewer#1, Concern # 4:** The Introduction could be updated with recent reviews dedicated to the references related to the topic addressed. In literature study the highlighted article might be considered (https://doi.org/10.1007/s11042-023-15363-4) for inclusion.

**Author response:** Thank you very much for your valuable comments. We added this literature (reference [10]), which is cited in the Introduction and Related Works sections, which is changed as follows.

…

Algorithms employed include Hidden Markov Model (HMM), Support Vector Machine, Decision Trees, Bayesian Networks, and Deep Neural Networks, among others [10].

…

Deep learning approaches can overcome some limitations of traditional shallow machine learning, capturing deep-seated features within the data, and enhancing detection performance [10].

[10] Dhasarathan C, Shanmugam M, Kumar M, Tripathi D, Khapre S, Shankar A. A nomadic multi-agent based privacy metrics for e-health care: a deep learning approach. Multimedia Tools and Applications. 2023; p. 1–24

**Reviewer#1, Concern # 5:** The English language should be more polished, and some typo corrections are required.

**Author response:** Thank you very much for your valuable comments. We greatly appreciate your feedback on the language of the article. We have carefully considered your suggestions to ensure a smoother and more polished language. Additionally, we have thoroughly reviewed the article and made necessary corrections for spelling errors to ensure a high-quality publication.

**Reviewer#1, Concern # 6:** There are different algorithm approaches currently in use. Why the author's selection towards the proposed system is preferred.

**Author response:** Thank you very much for your valuable comments. We believe that Hidden Markov Models (HMM) have extensive applications in intrusion detection, and, in comparison to other methods, HMM excels in its capacity to effectively model the stages. The alarm sequences align well with the HMM representation, as elaborated in the 'HMM' section of the 'Multi-stage profile' part of the manuscript. The details are as follows:

…

HMM is widely applied in attack detection, and literature[18-22] has provided substantial evidence of the effectiveness of HMM in attack detection. HMM has unique advantages in stage modeling, as alert sequence data can be effectively matched with the HMM representation. Therefore, this paper constructs MSP based on HMM.

**Reviewer#1, Concern # 7:** Rewrite or give reasonable explanations of notations used in equations.

**Author response:** Thank you very much for your valuable comments. In accordance with your advice, we have added Table 1, which serves as a symbol table to explain the formula symbols used in the paper. The specific details are reflected in the manuscript.

**Reviewer#1, Concern # 8:** List the limitations of the proposed system.

**Author response:** Thank you very much for your valuable comments. We have added a Future work section in the Conclusion section to explain the limitations of our proposed method. The details are as follows:

‘’‘

The method proposed in this paper can detect the presence of attack behavior and identify which alert information characterizes the attack. However, it cannot recognize the specific stage of the attack and reconstruct the attack scenario. Therefore, this method is not suitable for extracting attack scenarios. In future work, we will focus on more refined attack stage detection and attack scenario extraction.

**Reviewer#1, Concern # 9:** Describe the weakness of the proposed system.

**Author response:** Thank you very much for your valuable comments. We have added a Future work section in the Conclusion section to explain the limitations and weaknesses of our proposed method, as well as future research directions. The details are as follows:

…

The method proposed in this paper can detect the presence of attack behavior and identify which alert information characterizes the attack. However, it cannot recognize the specific stage of the attack and reconstruct the attack scenario. Therefore, this method is not suitable for extracting attack scenarios. In future work, we will focus on more refined attack stage detection and attack scenario extraction.

**Reviewer#2, Concern # 1:** However, I recommend revising the paper before proceeding further. References are shown as question marks (e.g., [?] on line 20 of page 2) and figures are numbered as "???" (e.g., Fig ?? on lines 35-36 of page 2). This makes it very difficult to understand which figures and references are being cited in the text.

**Author response:** Thank you very much for your valuable comments. We have reviewed our manuscript and corrected the issue of incorrect references, images, and table citations. The .tex file was compiled with latex miktex version 4.80 and the compiled .pdf file was checked with Microsoft Edge version 118.0 to make sure that no questions mark (which means unreachable reference) exists. We will submit the compiled PDF file for review when submitting the revised manuscript.

**Reviewer#3, Concern #1:** The key novel contribution in the proposed framework in Figure 2 should be well described, it looks like authors integrate the recent techniques together to form the framework.

**Author response:** Thank you very much for your valuable comments. We have rewritten the description of the framework proposed in Figure 2 and the main innovations are emphasized. The specific details are as follows:

…

The overall architecture of the method proposed in this paper, as shown in Fig 2, consists of four steps: network data acquisition (phase 1), alert preprocessing (phase 2), establishment of MSP (phase 3), and attack detection (phase 4).

Firstly, in phase 1, First, in Phase 1, we designed an automated data acquisition method to obtain alert information from network traffic. Deployed IDS in the network continuously analyze traffic data captured from the network environment and generate alerts when suspicious packets are detected based on predefined rules. IDS may not detect complete multi-stage attacks, but when attackers attempt to infiltrate through multiple attack stages, IDS may capture individual attack actions and issue corresponding alerts. In Phase 1, the system primarily faced performance pressure stemming from IDS traffic analysis, and therefore, we adopted an offline analysis strategy to avoid impacting the overall system performance.

However, it is worth noting that IDS systems generate a significant number of false positives. These alerts result from the inability of the alert generation rules to distinctly differentiate between normal and malicious activities within the network, and thus, do not represent genuine security threats[28]. Nevertheless, these non-attack stage alerts often carry information about the system's activity patterns, serving as a means to describe the system's normal state. Alerts generated by IDS are stored in the Alerts Database, which encompasses alert data from the system's normal state (referred to as non-attack stage alerts) and alert data from multi-stage attack states (referred to as attack stage alerts in this paper).

In phase 2, we introduce a method for alert preprocessing with the goal of transforming the text-style alert data generated in Phase 1 into data that can be used by machine learning algorithms. Since HMM cannot directly process alert information, we convert the alert data from the Alerts Database into vectors using the Doc2Vec algorithm. This allows us to extract deep-seated information from the alerts and analyze the associations between alert entries for further processing. The performance overhead in Phase 2 primarily stems from the training of the Doc2Vec model. Therefore, similar to Phase 1, we adopt an offline training strategy in Phase 2.

Then, the proposed MSP is constructed in Phase 3. We initially use a clustering approach to automatically obtain the stage division of normal alert vectors, which is then mapped to hidden states in the HMM to complete the construction of MSP based on HMM. Training the clustering model and HMM introduces a significant performance overhead in this phase. Therefore, similar to previous phases, we also employ an offline training approach.

Finally, in Phase 4, we perform online detection of alert data using the constructed MSP. The probability generated by the MSP is used as the basis for determining anomalies. This probability is compared to a predefined threshold to decide whether the sequence is anomalous. If it is, then the alert is marked as an attack stage alert. In contrast to Phases 1, 2, and 3, the detection process in Phase 4 is conducted online, using the MSP model obtained in the offline training of Phase 3. Online detection is nearly real-time, and it imposes relatively low performance overhead.

**Reviewer#3, Concern #2:** The performance impact of each stage in Figure 2 can be introduced and discussed.

**Author response:** Thank you very much for your valuable comments. The performance impact of each phase shown in Figure 2 are discussed in the modified manuscript. And we also conducted a series of experiments to validate our analysis, with the results illustrated in Figure 8. The specific details are reflected in the manuscript.

**Reviewer#3, Concern #3:** For the evaluation, the dataset/algorithm choosing reasons, the detailed platform configurations and the discussion on other untested datasets should be introduced in the revised paper.

**Author response:** Thank you very much for your valuable comments. We introduced the reasons for selecting datasets and algorithms, specific platform configurations, and reasons for not selecting other datasets in our paper. The details are as follows:

…

The datasets relevant to multi-stage attack detection include DARPA2000, ISCXIDS2012, CTU-13, DEFCON21 CTF, and CICIDS2017, among others. In these datasets, DARPA2000[35] contains specific scenarios with multi-stage attack samples, making it one of the most commonly used datasets in related research. Compared to two other datasets in the same series, DARPA1998 and DARPA1999, this dataset eliminates many generation errors and biases. It includes two attack scenarios, LLSDDOS1.0 and LLSDDOS2.0, with five attack stages. The ISCXIDS2012[36] dataset consists of real network traffic and encompasses complete multi-stage attacks with six stages, making it a common dataset for multi-stage attack detection research. The CICIDS2017[37] dataset is a supplement to the ISCXIDS2012 dataset and has also seen extensive use in recent related research. The data were collected continuously for five days from Monday to Friday and have been labeled.

…

The experimental setup for this study is as follows:

First, the experiments were conducted on a Windows 10 platform with an Intel(R) Core(TM) i5-7300HQ CPU @ 2.50GHz processor and 8GB of RAM. The development environment utilized Python 3.8, Keras 2.4.0, and TensorFlow 2.3.0.

Then, the network traffic data were replayed by Tcpreplay(version 4.2.6) and the alert texts were obtained by Snort(version 2.9.7) with default intrusion detection rules. The alert texts were interpreted with PV-DM by Doc2Vec before building MSP. Scikit-learn(version 1.0.2) and hmmlearn(version 0.2.3) were adopted in the process of building MSP. Hyper-parameters in the experiments are summarized in table 3.

…

HMM is widely applied in attack detection, and literature[18-22] has provided substantial evidence of the effectiveness of HMM in attack detection. HMM has unique advantages in stage modeling, as alert sequence data can be effectively matched with the HMM representation. Therefore, this paper constructs MSP based on HMM.

**Reviewer#3, Concern #4:** Some references lack the necessary information (e.g., [2]), please provide all information according to the right template.

**Author response:** Thank you very much for your valuable comments. We have reviewed our manuscript and made modifications according to the format required by the journal.

**Reviewer#3, Concern #5:** A notation table can be add to explain all math symbols.

**Author response:** Thank you very much for your valuable comments. In accordance with your advice, we have added Table 1, which serves as a symbol table to explain the formula symbols used in the paper. The specific details are reflected in the manuscript.

**Reviewer#3, Concern #6:** Please go through the paper carefully and double check whether the right template are used. Correct some typos and formatting issues (e.g., why all citation and figure refs do not display correctly).

**Author response:** Thank you very much for your valuable comments. We have reviewed our manuscript and corrected the issue of incorrect references, images, and table citations. We will submit the compiled PDF file for review when submitting the revised manuscript.

**Reviewer#3, Concern #7:** Make the References more comprehensive, besides this work, some other promising scenarios (e.g., Big data, other IoT systems) can be covered in this work. If the above related work can be discussed, it can strongly improve the research significance. For the improvement, the following papers can be considered to make the references more comprehensive.

Jin Wang, Changqing Zhao, Shiming He, Yu Gu, Osama Alfarraj, Ahed Abugabah, LogUAD: Log Unsupervised Anomaly Detection Based on Word2Vec, Computer Systems Science and Engineering, 2022, 41(3): 1207–1222

Kai Zhong, ZhiBang Yang, Guoqing Xiao, Xingpei Li, Wangdong Yang, Kenli Li: An Efficient Parallel Reinforcement Learning Approach to Cross-Layer Defense Mechanism in Industrial Control Systems. IEEE Trans. Parallel Distributed Syst. 33(11): 2979-2990 (2022)

J. Wang, Y. Yang, T. Wang, R. Sherratt, J. Zhang. Big Data Service Architecture: A Survey. Journal of Internet Technology, 2020, 21(2): 393-405

J. Zhang, R. Zhang, O. Alfarraj, A. Tolba, G.-J. Kim. A Memory-Aware Spark Cache Replacement Strategy. Journal of Internet Technology, 2022, 23(6): 1185-1190

Bin Pu, Kenli Li, Shengli Li, Ningbo Zhu: Automatic Fetal Ultrasound Standard Plane Recognition Based on Deep Learning and IIoT. IEEE Trans. Ind. Informatics 17(11): 7771-7780 (2021)

Lixia Ji, Xiao Zhang, Yao Zhao, Zongkun Li, "Anomaly Detection of Dam Monitoring Data based on Improved Spectral Clustering," Journal of Internet Technology, vol. 23, no. 4 , pp. 749-759, Jul. 2022.

**Author response:** Thank you very much for your valuable comments. We have carefully considered your suggestion and cited four of the six references you proposed (references [23] [24] [32] [34]). The specific details are as follows:

…

Furthermore, With the advancement of big data technology [23], some deep learning methods have been applied to multi-stage attack detection. Deep learning approaches can overcome some limitations of traditional shallow machine learning, capturing deep-seated features within the data [24], and enhancing detection performance.

[23] Wang J, Yang Y, Wang T, Sherratt RS, Zhang J. Big data service architecture: a survey. Journal of Internet Technology. 2020;21(2):393–405.

[24] Pu B, Li K, Li S, Zhu N. Automatic fetal ultrasound standard plane recognition based on deep learning and IIoT. IEEE Transactions on Industrial Informatics. 2021;17(11):7771–7780.

…

Word2Vec [32] addresses the dimensionality issue but loses sequence information by averaging word vectors. When using Word2Vec to compute text similarity, keyword extraction algorithms may not perform accurately.

[32] Wang J, Zhao C, He S, Gu Y, Alfarraj O, Abugabah A. LogUAD: log unsupervised anomaly detection based on Word2Vec. Computer Systems Science and Engineering. 2022;41(3):1207.

…

Basically, similar alerts are positioned close to each other in the vector space, making it possible to utilize clustering algorithms to assist HMM modeling. Clustering algorithms are widely employed in anomaly detection [34].

[34] Ji L, Zhang X, Zhao Y, Li Z. Anomaly Detection of Dam Monitoring Data based on Improved Spectral Clustering. Journal of Internet Technology. 2022;23(4):749–759
