# Supplementary material for: Anomaly based multi-stage attack detection method
Source: PLoS One. 2024 Mar 25;19(3):e0300821. doi: 10.1371/journal.pone.0300821 (PMC10962843; doi:10.1371/journal.pone.0300821)
Supplement: Attachment — Submitted filename: Response to Reviewers.docx [file pone.0300821.s002.docx]

**Original Article Title: “Anomaly based multi-stage attack detection method**”

**R****eviewer#4, Concern # 1:** The statistical analysis has not been conducted appropriately and rigorously.

**Author response:** Thank you very much for your valuable comments. We primarily employ the statistical method of 'representing probabilities in terms of frequencies.' This involves using statistical frequencies to substitute for Hidden Markov Model (HMM) initial probabilities and transition probabilities. This is a commonly used data statistical technique, and similar applications can be found in other literature. For instance, Zhang et al. [1] and Shawly et al. [2] have both utilized this approach in their respective works.

**Reviewer#4, Concern # 2:** The author has not provided all the data on which the manuscript relies.

**Author response:** Thank you very much for your valuable comments. All the data used in this paper have been sourced from public datasets, and the download link for the dataset has been provided in the references and on the Plos One submission page. Readers have the option to download the dataset from the website and replay the downloaded .pcap data through Tcpreplay 4.2.6. Snort 2.9.7 (utilizing the default Snort rules) has been employed to detect the replayed traffic and generate alarm data.

**Reviewer#4, Concern # 3:** There are a lot of assumption that cannot be accepted. Results depends on the ruleset used in Snort, which it is not identified in the paper. Only a Little part of multistage attacks can be detected With network traffic(around 30% of mittre att&ck attack matrix attacks). There are so many attacks that are not detected in Snort rules (depending on ruleset configuration).

**Author response:** Thank you for your valuable comments while reviewing my paper. The following are detailed answers to your questions.

First of all, the hypotheses we put forward in the paper are as follows:

1. If the contexts described by two alarms are similar, then they can be considered to have similar semantics.

2. The process of multi-stage attack from alarm clustering to each cluster conforms to homogeneous Markov property.

For the first hypothesis, we are based on the "context similarity principle", which means that similar contexts correspond to similar meanings. This assumption has also been used in literature [3]. For the second assumption, clusters after alarm clustering have similar behavior patterns or characteristics, and these clusters can be regarded as different states in the system, and this similarity is similar to the similarity between adjacent states in the Markov chain, which has the same assumption in literature [1]. These assumptions above are made mainly to simplify the model and make the experiment feasible.

Secondly, the Snort rules used in the experiments of this paper are all default rules, and we have added "default rules were used" in the manuscript.

Finally, you mentioned that only a small number of multi-stage attacks would be detected by Snort. However, in order to eliminate the impact of different Snort rules on detection and enhance the reproducibility and comparability of research, the methodology of multi-stage attack detection research has always used the alarm data generated by Snort default rules as the detection data source. For example, "Applications of Hidden Markov Models to Detecting Multi-stage Network Attacks" proposed by Ourston et al. [4] in 2003, In 2005 Mathew et al. [5] proposed "Real-time multistage attack awareness through enhanced intrusion alert clustering", "Real Time Alert Correlation and Prediction using Bayesian Networks" proposed by Ramaki et al. [6] in 2015, "Real-time multistep attack prediction based on Hidden Markov Models" proposed by Holgado et al. [7] in 2020, In 2020, Shawly et al. [2] proposed "Evaluation of HMM-Based Network Intrusion Detection System for Multiple Multi-Stage Attacks", "Multi-Stage Attack Detection via Kill Chain State Machines" proposed by Wilkens et al. [8] in 2021, In 2022, Zhang et al. [1] proposed "Multi-Step Attack Detection Based on Pre-Trained Hidden Markov Models", etc.

Thank you again for your modification suggestions. At the same time, writing more insightful Snort rules is also a direction of our future research, and we will focus on improving the detection effect in terms of data source quality in the future research.

**References**

[1] Zhang, X., Wu, T., Zheng, Q., Zhai, L., Hu, H., Yin, W., ... & Cheng, C. (2022). Multi-step attack detection based on pre-trained hidden Markov models. *Sensors*, *22*(8), 2874.

[2] Shawly, T., Khayat, M., Elghariani, A., & Ghafoor, A. (2020). Evaluation of hmm-based network intrusion detection system for multiple multi-stage attacks. *IEEE Network*, *34*(3), 240-248.

[3] Zhou, P., Zhou, G., Wu, D., & Fei, M. (2021). Detecting multi-stage attacks using sequence-to-sequence model. *Computers & Security*, *105*, 102203.

[4] Ourston, D., Matzner, S., Stump, W., & Hopkins, B. (2003, January). Applications of hidden markov models to detecting multi-stage network attacks. In *36th Annual Hawaii International Conference on System Sciences, 2003. Proceedings of the* (pp. 10-pp). IEEE.

[5] Mathew, S., Britt, D., Giomundo, R., Upadhyaya, S., Sudit, M., & Stotz, A. (2005, October). Real-time multistage attack awareness through enhanced intrusion alert clustering. In *MILCOM 2005-2005 IEEE Military Communications Conference* (pp. 1801-1806). IEEE.

[6] Ramaki, A. A., Khosravi-Farmad, M., & Bafghi, A. G. (2015, September). Real time alert correlation and prediction using Bayesian networks. In *2015 12th International Iranian Society of Cryptology Conference on Information Security and Cryptology (ISCISC)* (pp. 98-103). IEEE.

[7] Holgado, P., Villagrá, V. A., & Vazquez, L. (2017). Real-time multistep attack prediction based on hidden markov models. *IEEE Transactions on Dependable and Secure Computing*, *17*(1), 134-147.
