# Supplementary material for: Anomaly based multi-stage attack detection method
Source: PLoS One. 2024 Mar 25;19(3):e0300821. doi: 10.1371/journal.pone.0300821 (PMC10962843; doi:10.1371/journal.pone.0300821)
Supplement: Attachment — Submitted filename: Response to Reviewers.docx [file pone.0300821.s003.docx]

**Original Article Title: “Anomaly based multi-stage attack detection method**”

**Reviewer#4, Concern # 1:** In fact, for web traffic, the article [X] presents experimental work demonstrating that only a small percentage of web attacks (around 10%) are caught by Snort with default rules. While it is true that this percentage rises sharply to 80% when additional rules are employed, it is also true that the number of false positives generated reduces accuracy from values close to 100% with default rules to 1.5%. Therefore, the authors are requested to include in their work this caveat, suggesting for future research that it would be beneficial to experiment with different rule sets and consider the false positives they generate in legitimate traffic.

**Author response:** Thank you for your valuable comments while reviewing my paper. We have incorporated the following content into the future work section of our paper as per your suggestion. The detailed changes are as follows:

…

Furthermore, the selection of alert information generated by Snort has been a consistent methodology. However, it should be noted that Snort does not have the ability to detect all attack behaviors and generate alert information. The detection capability of Snort is highly dependent on the rules used. Therefore, in our future research, we will attempt to use different sets of Snort rules and consider their false positives in legitimate traffic.

In the research on multi-stage attack detection, most researchers have chosen Snort default rules to generate alert information for different datasets[8][13][17], making their methods reproducible and easy to compare. In fact, for network traffic, Snort's default rules can only capture a small portion of attacks (10\%), and when additional rules are used, the proportion of captured attacks increases to 80\%, but this also reduces the accuracy from nearly 100\% with default rules to 1.5\%. Hence, another task for our future endeavors is to develop more insightful Snort rules, aiming to enhance data quality and, consequently, improve detection accuracy.

…
